# A Preliminary Investigation of the Association of Sleep With Inflammation and Oxidative Stress Biomarkers and Functional Outcomes After Stroke Rehabilitation

**DOI:** 10.1038/s41598-017-08931-w

**Published:** 2017-08-17

**Authors:** Ting-ting Yeh, Yu-wei Hsieh, Ching-yi Wu, Jong-shyan Wang, Keh-chung Lin, Chia-ling Chen

**Affiliations:** 1 School of Occupational Therapy, College of Medicine, National Taiwan University, 17, F4, Xu Zhou Road, Taipei, Taiwan; 2 Department of Occupational Therapy and Graduate Institute of Behavioral Sciences, College of Medicine, Chang Gung University, 259 Wenhua 1st Rd., Guishan Dist., Taoyuan City, Taiwan; 3 Healthy Aging Research Center, Chang Gung University, 259 Wenhua 1st Rd., Guishan Dist., Taoyuan City, Taiwan; 4 Department of Physical Medicine and Rehabilitation, Chang Gung Memorial Hospital at Linkou, 5 Fuxing St., Guishan Dist., Taoyuan City, Taiwan; 5 Graduate Institute of Rehabilitation Science, Chang Gung University, 259 Wenhua 1st Rd., Guishan Dist., Taoyuan City, Taiwan; 6 Division of Occupational Therapy, Department of Physical Medicine and Rehabilitation, National Taiwan University Hospital, 7 Chung Shan South Rd., Taipei, Taiwan; 7 Graduate Institute of Early Intervention, College of Medicine, Chang Gung University, 259 Wenhua 1st Rd., Guishan Dist., Taoyuan City, Taiwan

## Abstract

This study examined the association of sleep with inflammation and oxidative stress biomarkers, and with functional outcomes, after stroke rehabilitation. The rehabilitation effects on biomarkers and functional outcomes were also evaluated. Twenty subacute stroke survivors received 4 weeks of upper limb rehabilitation. Baseline inflammatory (i.e., soluble intercellular adhesion molecule-1, sICAM-1) and oxidative stress biomarkers (i.e., glutathione peroxidase, GPx and malondialdehyde, MDA) were assessed, as were sleep outcomes. Positive correlations were observed between baseline level of sICAM-1 and number of awakenings at post-treatment (ρ = 0.51, *p* < 0.05) as well as between baseline level of MDA and post-performance time of the Wolf Motor Function Test (WMFT-time) (ρ = 0.46, *p* < 0.05). In addition, MDA levels were significantly decreased, and functional outcomes of the modified Rankin Scale (mRS), functional ability scale of the WMFT, and Stroke Impact Scale (SIS-total, and SIS-physical function) were improved after the rehabilitation. This pilot study emphasizes the relationship among biomarkers, sleep, and functional outcomes after stroke rehabilitation. Oxidative stress markers may be useful predictors of functional outcomes in subacute stroke survivors.

## Introduction

Stroke, a leading cause of long-term disability, produces immense health and economic burdens globally^1^. Over the last two decades, the relationship between stroke and sleep disorders has been of growing interest to researchers and practitioners^2, 3^. Sleep disorders are found in up to 78% of stroke survivors^4^. These sleep disorders, including sleep apnea, insomnia, hypersomnia, and parasomnia, interfere with normal physical, mental, social, and emotional functioning^5^. Sleep is associated with a patient’s ability to achieve the full potential for stroke recovery^6^; therefore, untreated sleep disorders may diminish rehabilitation efforts and result in poor functional prognosis.

Evidence suggests that sleep disorders are an independent factor that predicts functional disability and longer hospitalization following stroke^7^. Specifically, Kaneko *et al*. found that stroke survivors with sleep apnea had lower functional ability, as measured by the Functional Independence Measure (FIM), than did those without sleep apnea^7^. A more recent study also suggests that sleep disturbances in stroke survivors can detrimentally affect functional status, such as scores on the modified Barthel Index and grip strength^8^.

The existing literature describing the relationships between sleep disorders and biomarkers, such as inflammatory and oxidative stress pathways, is extensive^9, 10^. A meta-analysis of 51 studies found that, compared to control subjects, patients with obstructive sleep apnea (OSA) have higher levels of inflammatory markers such as C-Reactive protein (CRP), tumor necrosis factor alpha (TNF-α), and intercellular adhesion molecule (ICAM)^10^. These levels are positively correlated to the level of OSA severity^10^. On the other hand, previous studies have suggested that elevated inflammatory and oxidative markers may be associated with the pathogenesis of cardiovascular and cerebrovascular diseases^10–12^. The underlying mechanisms may involve endothelial dysfunction, systemic inflammation, oxidative stress, and metabolic dysregulation resulting from hypoxemia, reoxygenation, and intrathoracic pressure^13^. Increased production of pro-inflammatory cytokines has been observed in stroke survivors and may be associated with poor stroke outcomes. Specifically, elevated cytokines, such as interleukin-6, presented in the early stroke period are closely associated with worse stroke outcomes assessed by the Barthel Index and modified Rankin scale^14^. Another study showed that reduced levels of inflammation are negatively correlated with the Barthel Index, suggesting that the decrease of inflammatory markers was beneficial for stroke recovery^15^. In addition, our previous work demonstrated that the changes of patients in oxidative stress level, measured by 8-hydroxy-2′-deoxyguanosine, were significantly correlated with the upper limb motor function, muscle power, and perception of pain^16^. Disturbed sleep is associated with an increased level of inflammation^10^. There is also evidence suggesting that systemic inflammation results in a disruption of sleep^17, 18^. Chronic inflammation may reduce sleep continuity and depth and induce sleep disorder, such as insomnia, suggesting a relationship between disturbed sleep and inflammation^19^. Therefore, examining the associations among level of inflammation, level of oxidative stress, and sleep outcomes is particularly relevant after stroke, given that changes in any one of these factors are proposed to be related to functional prognosis and the effectiveness of rehabilitation treatment post-stroke^20, 21^.

Recent work by Chen and colleagues^22^ demonstrated that the severity of OSA is positively associated with the total antioxidant capacity (TAC) and CRP in ischemic stroke subjects. Specifically, the results of the study showed that the levels of CRP and TAC were positively correlated with the oxyhemoglobin desaturation index; the TAC levels were negatively correlated with mean arterial oxygen saturation in stroke survivors with severe OSA^22^. However, what has been lacking to date is examination of the relationships among the biomarkers, sleep, and functional and health-related outcomes after stroke rehabilitation.

Stroke rehabilitation programs, such as robot-assisted therapy^23, 24^ and task-oriented training^25, 26^, may improve functional outcomes in stroke survivors. Research supports the efficacy of upper limb robot-assisted therapy for improving motor and functional outcomes in stroke patients^23, 24^. Moreover, task-oriented training with stroke survivors improves functional outcomes and overall health-related quality of life^25, 26^. A better understanding of the changes of the inflammation, oxidative stress, and sleep indicators following stroke rehabilitation is crucial not only for predicting treatment-related outcomes but also for monitoring clinical response and adverse effects.

The pilot study aimed to: (1) characterize the relationships among baseline biomarkers (i.e., inflammation and oxidative stress), sleep, and functional and health-related outcomes at post-treatment in subacute stroke patients, and (2) investigate the changes in levels of biomarkers, sleep, and functional and health-related outcomes after 4 weeks of rehabilitation intervention. We hypothesized that (1) higher levels of inflammation and oxidative stress at baseline would be associated with poor sleep quality and functional and health-related outcomes post-treatment, and (2) the levels of inflammation and oxidative stress would decrease, and the functional and health-related outcomes would improve, after the intervention.

## Results

### Baseline characteristics

The study enrolled a total of 20 patients with subacute stroke. Table 1 summarizes the patients’ demographic and baseline clinical characteristics. The mean age was 48.17 years, and the ratio of males to females was 1:1. Functional impairment and level of disability, as measured by self-report and performance-based measures, were generally moderate. Individuals with and without sleep disturbances, based on the global Pittsburg Sleep Quality Index (PSQI) score, and the patient characteristics are also included. Among these patients, fifteen were characterized as bad sleepers (i.e., global PSQI score > 5). The ratio of good sleepers to bad sleepers was 1:3.

**Table 1:** Demographics and baseline clinical characteristics of the participants.

Characteristics*	All (N = 20)	Good sleeper (PSQI score ≤ 5, *n* = 5)	Bad sleeper (PSQI score > 5, *n* = 15)
Age (y)	48.17 ± 10.59	54.74 ± 9.91	45.97 ± 10.17
Time after stroke (mo)	2.46 ± 1.34	2.51 ± 0.77	2.44 ± 1.51
Sex			
Male	10	3	9
Female	10	2	6
Side of stroke			
Right	10	4	9
Left	10	1	6
Stroke type			
Ischemic	8	1	7
Hemorrhagic	12	4	8
MMSE score	26.75 ± 2.61	25.6 ± 3.85	27.13 ± 2.10
PSQI total score (0–21)	6.6 ± 2.19	3.8 ± 0.84	7.53 ± 1.60
Sleep percent	41.87 ± 6.66	45.50 ± 6.79	41.92 ± 6.95
Number of awakenings	88.53 ± 35.26	67.75 ± 15.12	94.47 ± 37.50
WMFT-time (0–120 s)	11.44 ± 8.84	11.32 ± 6.39	11.49 ± 9.72
WMFT-FAS (0–5)	2.22 ± 0.89	2.61 ± 0.92	2.09 ± 0.87
SIS-total (0–100)	59.37 ± 13.19	58.65 ± 16.48	59.62 ± 12.54
SIS-physical function	46.42 ± 17.14	45.87 ± 16.26	46.61 ± 17.97
SIS-stroke recovery	52 ± 19.29	61 ± 14.32	49 ± 17.97
mRS (0–5)	3.25 ± 0.85	3.2 ± 1.10	3.27 ± 0.80

MMSE = Mini-Mental State Examination; PSQI = Pittsburg Sleep Quality Index; WMFT-time = performance time of the Wolf Motor Function Test; WMFT-FAS = functional ability scale of the Wolf Motor Function Test; SIS = Stroke Impact Scale; mRS = modified Rankin Scale. *Continuous data are presented as the mean ± SD; categorical data are presented as indicated.

### Association between inflammation, oxidative stress markers, sleep and functional outcomes

The Spearman rank correlation coefficient revealed that the pre-sICAM-1 was positively correlated with the post-number of awakenings (ρ = 0.506, *p* < 0.05) (Table 2), indicating that patients with higher baseline levels of inflammation had higher numbers of awakenings at post-treatment. However, the pre-sICAM-1 level had no statistically significant correlations with other sleep and functional outcomes (all *p*’s > 0.05). In addition, there was a significant positive correlation between pre-MDA and post-WMFT-time (ρ = 0.462, *p* < 0.05), and an almost significant correlation between pre-MDA and post-mRS (ρ = 0.421, *p* = 0.065). However, pre-GPx was not correlated with any post-sleep or post-functional outcomes (all *p*’s > 0.05).

**Table 2:** Spearman correlation coefficient (ρ) between baseline biomarkers and post-treatment sleep and functional outcomes (N = 20).

	Sleep (post)			Functional outcomes (post)					
	PSQI	Sleep percent	Number of awakenings	mRS	WMFT-time	WMFT-FAS	SIS	SIS-Physical function	SIS-Stroke recovery
*pre*-sICAM-1	0.133	0.355	0.506*	−0.239	−0.248	0.191	0.254	0.251	0.204
*pre*-GPx	0.242	0.119	0.032	−0.281	−0.071	0.041	0.021	0.029	−0.153
*pre*-MDA	<0.001	0.212	0.183	0.421^†^	0.462*	−0.06	−0.381	−0.365	−0.221

sICAM-1 = Soluble intercellular adhesion molecule-1; GPx = Glutathione peroxidase; MDA = Malondialdehyde; PSQI = Pittsburg Sleep Quality Index; mRS = modified Rankin Scale; WMFT-time = performance time of the Wolf Motor Function Test; WMFT-FAS = functional ability scale of the Wolf Motor Function Test; SIS = Stroke Impact Scale.

^*^ *p* < 0.05, ^†^ *p* = 0.065.

### Changes in biomarkers, sleep, and functional outcomes from pre-treatment to post-treatment

The pre- and post-treatment levels of biomarkers, sleep outcomes, and functional outcomes are reported in Table 3. For the inflammation and oxidative stress markers, only the MDA demonstrated a significant decrease from pre- to post-treatment (z = 2.54, *p* < 0.05), indicating lower oxidant levels of the patients after treatment. No significant pre- or post-treatment changes were noted for the PSQI total score, sleep percentage, and number of awakenings (all *p*’s > 0.05). However, there was a trend of improvement for biomarkers and sleep outcomes from pre-treatment to post-treatment. Furthermore, after treatment, patients improved significantly on most of the functional outcomes. Specifically, significant changes from pre- to post-treatment were found on the mRS (z = 3.45, *p* = 0.001), WMFT-FAS (z = −3.72, *p* < 0.001), SIS-total (z = −2.98, *p* = 0.003), and SIS-physical function (z = −3.38, *p* = 0.001).

**Table 3:** Inflammation, oxidative stress levels, sleep and functional and health-related outcomes before and after treatment in patients with stroke (N = 20).

Outcome	Pre-treatment (Mean ± SD)	Post-treatment (Mean ± SD)	*P* value
Inflammatory and oxidative markers			
sICAM-1	275.34 ± 80.38	258.48 ± 74.97	0.55
GPx	36.17 ± 11.47	37.66 ± 11.34	0.48
MDA	4.02 ± 1.26	3.55 ± 1.08	0.01*
Sleep			
PSQI total score (0–21)	6.6 ± 2.19	6.3 ± 2.66	0.68
Sleep percent (actigraph)	41.87 ± 6.66	43.01 ± 7.07	0.26
Number of awakenings (actigraph)	88.53 ± 35.26	79.55 ± 38.01	0.32
Functional and health-related outcomes			
mRS (0–5)	3.25 ± 0.85	2.35 ± 0.99	0.001*
WMFT-time (0–120 s)	11.44 ± 8.84	10.40 ± 6.48	0.58
WMFT-FAS (0–5)	2.22 ± 0.89	2.69 ± 1.03	<0.001*
SIS-total (0–100)	59.37 ± 13.19	65.27 ± 13.81	0.003*
SIS-physical function	46.42 ± 17.14	56.75 ± 19.56	0.001*
SIS-stroke recovery	52.00 ± 19.29	55.25 ± 19.50	0.35

sICAM-1 = Soluble intercellular adhesion molecule-1; GPx = Glutathione peroxidase; MDA = Malondialdehyde; PSQI = Pittsburg Sleep Quality Index; mRS = modified Rankin Scale; WMFT-time = performance time of the Wolf Motor Function Test; WMFT-FAS = functional ability scale of the Wolf Motor Function Test; SIS = Stroke Impact Scale; SD = standard deviation. *p < 0.05.

## Discussion

This study aimed to characterize the relationships of baseline inflammation and oxidative stress biomarkers to sleep and to functional and health-related outcomes after 4 weeks of rehabilitation intervention in subacute stroke patients. First of all, a positive association was observed between the baseline level of inflammation and the number of awakenings at post-treatment. We also found a positive correlation between the baseline oxidant level and post-treatment upper limb motor function (i.e., the post-WMFT-time). A similar trend was found for baseline oxidant level and post-treatment level of stroke disability (i.e., the post-mRS). In addition, significant effects were decreased oxidant levels and improved motor function and health-related outcomes after the rehabilitation. Although not statistically significant, there was a trend suggesting promising and beneficial rehabilitation intervention effects on the biomarkers and sleep outcomes.

The present study expands on previous findings by showing the association between inflammation markers and objectively measured sleep outcomes in stroke survivors. Elevated levels of inflammation have been found to predict an increased risk for adverse health outcomes such as myocardial infarction and diabetes in healthy populations^27, 28^. In this study, increases in the baseline level of the inflammatory sICAM-1 were found to be associated with higher numbers of awakenings at post-treatment. This result may support the hypothesis that high inflammation at baseline is related to poor sleep quality via the worsening of comorbidities associated with inflammation. This finding is partly consistent with previous findings that higher levels of inflammation reduce non-rapid-eye-movement sleep due to diminished sleep-promoting effects, and thereby increase wakefulness^29^. Many studies have found that a sleep deficiency induces systematic inflammation, which may be associated with metabolic changes and immunological deregulation^29–31^. This study, on the other hand, suggests a reciprocal regulation between inflammatory response and sleep loss. Our finding of a link between higher baseline inflammation and poor sleep quality echoes previous work suggests that pro-inflammatory cytokines induce sickness symptoms such as sleepiness, fatigue, and poor cognition^32^.

Oxidative stress has been found to play an important role in acute ischemic stroke pathogenesis. Ozkul *et al*. investigated serum MDA levels in patients with acute stroke and found a negative correlation with clinical outcomes measured by the Canadian Neurological Scale^33^. Although elevated oxidative stress has been reported in stroke patients, few studies have described the correlation between this biomarker and functional outcomes after stroke rehabilitation. Hsieh *et al*. demonstrated that urinary 8-hydroxy-2′-deoxyguanosine (8-OHdG), a biomarker of oxidative DNA damage, is a valid predictor of functional outcomes in chronic stroke survivors. Specifically, they showed that the baseline 8-OHdG content was significantly correlated with post-treatment functional outcomes such as the Fugl-Meyer Assessment (FMA) and Medical Research Council scale (MRC)^16^. In the present study, we measured the MDA level as a marker of oxidative stress and observed that baseline serum MDA levels were significantly correlated with post-treatment motor function measured by the WMFT-time. This relationship may not be attributed to the influence of treatment because WMFT-time scores did not change significantly from pre- to post-treatment. A higher level of baseline MDA content was significantly associated with less favorable functional outcomes (i.e., WMFT-time) after rehabilitation. This finding indicated that the oxidative stress markers might be valid in predicting functional outcomes in stroke survivors. Our results also found a similar trend for baseline oxidant level and post-treatment level of disability measured by the mRS. However, we did not find any significant correlations between antioxidants (i.e., GPx) and post-treatment functional outcomes.

Another key finding is that the 4 weeks of rehabilitation produced significant effects on oxidative stress levels, indicating that the intervention, did not produce oxidative stress in the stroke survivors, but rather alleviated it. In addition, some of the functional and health-related outcomes were significantly improved after the intervention as compared with the baseline. Rehabilitative intervention such as robot-assisted therapy is considered to be an intervention approach for restoring upper limb function and has been examined in large clinical trials in stroke patients^34, 35^. Regular rehabilitative exercise including functional and movement training has been shown to promote upregulation of antioxidant capacity and attenuate oxidative stress in stroke patients^36^. Although our results showed a trend of increasing antioxidant capacity, the significantly decreased oxidative stress may suggest the possibility of the reduced occurrence of oxidative damage, which may partially contribute to improving functional and health-related outcomes.

Sleep disorders, which are common in stroke patients, are associated with poorer stroke recovery outcomes and increased cerebrovascular morbidity. It has been shown that, in stroke survivors, better sleep quality is associated with better functional status^8^ and cognitive recovery^37^. Although not designed for this purpose, we noted that stroke survivors with good sleep quality at baseline had lower oxidant levels and greater health-related outcomes (i.e., the SIS total score) after the treatment. All participants had improved motor function and physical function, but only the bad sleepers showed decreased level of disability following the treatment. Individuals with sleep disturbance had lower level of disability at baseline and may have more room for improvement. Of note, a limitation of the study was the relatively small sample size, especially the good sleepers. Follow-up study using a larger sample for study is needed to validate the findings. In addition, we used a subjective measure, the PSQI, to quantify sleep quality and differentiated between good and bad sleepers. Further research employing an objective sleep monitoring system, such as actigraphy, to record the quality, quantity, and rhythm of sleep may provide better understanding of the relationship of sleep outcomes to functional recovery after stroke. Also, future work could include other potential inflammatory and oxidative stress biomarkers, such as CRP or TAC, to establish a strong correlation between biomarkers of oxidative stress and functional outcomes. Finally, future work could also examine different rehabilitative approaches that activate the biochemical regulations most likely to promote neuronal plasticity and recovery following stroke.

## Methods

### Participants

Patients with subacute stroke were recruited if they met the following criteria: (1) unilateral stroke with onset of less than 6 months; (2) upper extremity score of the Fugl-Meyer Assessment (FMA) > 10; (3) no excessive spasticity in any joints of the affected arm (modified Ashworth Scale ≤ 3); (4) ability to follow study instructions and perform tasks (Mini-Mental State Examination Score ≥ 22); (5) no other neurologic or major orthopedic diseases. All participants signed informed consent forms approved by the institutional review boards. Ethical approval was provided by the Institutional Review Board for Human Studies of Chang Gung Memorial Hospital. All methods were performed in accordance with the relevant guidelines and regulations.

### Stroke rehabilitation intervention

The stroke rehabilitation programs included robot-assisted therapy and task-oriented approach training. All patients received one 90-minute therapy session each day, 5 days per week, for 4 weeks. The training sessions were delivered by certified occupational therapists, and the daily logs of therapeutic activities were recorded.

Robot-assisted therapy was carried out using the Bi-Manu-Track device (Reha-Stim Co, Berlin, Germany) for bilateral arm symmetrical training^24, 38^^.^ This robotic device delivers forearm pronation/supination and wrist flexion/extension movements. Each movement pattern has 3 modes: (1) passive-passive, (2) active-passive, and (3) active-active. In the passive-passive mode, the robot controls movements of both arms. In the active-passive mode, either the affected or non-affected arm moves the robot handle while the other robot handle moves the non-affected or affected arm at the same movement speed and amplitude. In the active-active mode, both arms perform movements by overcoming an initial isometric resistance. The movement speed, amount of resistance, and range of movement can be adjusted individually. In this study, each participant performed an average of 1,200 to 1,600 movement repetitions for each session.

During the task-oriented training, participants practiced functional activities in accordance with their functional levels and individual goals. Examples of the functional activities are as follows: (1) walk to a water fountain, fill a glass with water, and drink; (2) wring out a wet rag and wipe a Table 3 fold towels and walk to a drawer and put the towels in it. The therapist used task-oriented approach principles and promoted motor function by increasing the complexity of the task over time. The therapist encouraged the patient to use the affected arm to execute tasks and then provided feedback to facilitate performance during training.

### Outcome measures

All measurements were obtained at baseline (pre-treatment) and 4 weeks after treatment (post-treatment).

### Sleep outcomes

Self-reported sleep disturbances were evaluated using the Pittsburgh Sleep Quality Index (PSQI), which includes 7 components: subjective sleep quality, latency, duration, efficiency, sleep disturbances, use of sleep medication, and daytime somnolence. The component scores are summed to produce a global score (ranging from 0 to 21). Higher scores indicate worse sleep quality. A global PSQI score greater than 5 indicates poor sleepers^39^. In addition to the subjective measurement of sleep outcome, each participant wore an accelerometer to detect and record arm movements. The data collected from the accelerometers were used as a proxy for assessment of sleep and wake. The sleep percentage (total sleep time divided by total wearing time) and the total number of awakenings for 3 consecutive days at baseline and 4 weeks post-treatment (3 valid days were required for analysis) were collected using the MicroMini-Motionlogger activity monitor (Ambulatory Monitoring, New York, NY, USA). The accelerometer was set to record hand movement in 60-sec epochs using the Proportional Integrating Mode (PIM) that captures the amplitude of arm movement. The data were further configured using ACT-Millennium software (AMI) and processed in Action4 software. The accelerometer was worn on the non-affected wrist. The accelerometer was removed during the 90-minute therapy session; therefore, the average total wearing time for each participant was approximately 22 to 22.5 hours per day. While wearing the accelerometer, participants completed daily monitoring logs to document the activities they performed (e.g., walking, watching TV, reading…etc.), the time they went to bed, and periods when the accelerometer was removed. The accelerometer daily monitoring log was used to identify any discrepancy between self-reported functional use of the non-affected arm and the accelerometric data.

### Inflammation and oxidative stress biomarkers

We evaluated the level of the soluble intercellular adhesion molecule-1 (sICAM-1) as the pro-inflammatory marker. Elevated concentrations of sICAM-1 are associated with local or systemic inflammation^40^. The evaluated oxidative stress indicators included glutathione peroxidase (GPx) and malondialdehyde (MDA). GPx is an antioxidant enzyme that can be used to evaluate the body’s defense mechanism against oxidative stress indicators. MDA is a biomarker for tissue damage and reperfusion^41^. A higher level of GPx indicates a higher antioxidant level, whereas a higher level of MDA reflects a higher oxidant level. Standard operating procedures consistent with expert consensus recommendations were used to collect, process, and store blood samples^42^. We performed the measurements using colorimetric enzyme-linked immunosorbent assay (ELISA) following the manufacturer’s instructions for sICAM-1 (BioLegend, San Diego, CA, USA) and MDA (MDA-586, OxisResearch, Portland, OR, USA). Level of GPx was evaluated using the Glutathione Peroxidase assay kit (Cayman Chemical Company, Ann Arbor, MI, USA). The units of measurement are expressed as ng/ml for sICAM-1, nmol/min/ml for GPx, and μM for MDA.

### Functional and health-related outcomes

We used the modified Rankin Scale (mRS), Wolf Motor Function Test (WMFT), and Stroke Impact Scale (SIS) to evaluate level of stroke disability, motor function, and health outcomes, respectively. Level of stroke disability related to activity performance and participation was assessed using the mRS. The mRS is a six-point ordinal scale commonly used in stroke patients^43^. The mRS score ranges from 0 to 5, with 0 representing “no symptoms” and 5 representing “severe disability.” A change of one or more points indicates meaningful improvement or decline in functional independence over time^44^. Its validity and inter-rater reliability are well documented^45^. The WMFT is a laboratory-based measurement for assessing upper-extremity motor function^46^. The WMFT comprises 17 tasks with 15 function-based and 2 strength-based tasks. We calculated two different scores: the WMFT-time (time required to complete the tasks, with a maximum of 120 s) and WMFT-FAS (Functional Ability Scale, which is used to assess the functional capacity of each task; 0 indicates that the patient cannot attempt the task, and 5 indicates that the movement appears normal). The psychometric properties of the WMFT have been validated in patients with stroke^47, 48^. The SIS is a stroke-specific, self-report, and health status measure that assesses multidimensional stroke outcomes^49^. Items are scored on a Likert-type scale of 1 (inability to complete the item) to 5 (no difficulty at all). We collected data from all domains and generated a global score (SIS-total). The subdomains in physical function (SIS-physical function) and ratings on overall stroke recovery (SIS-stroke recovery) were also reported.

### Statistical analysis

We used the Spearman rank correlation coefficient to quantify the correlations among the pre-treatment biomarkers (e.g., inflammation and oxidative stress), sleep, and functional and health-related outcomes at post-treatment. We also performed a Wilcoxon signed rank test to examine the statistical significance of changes from pre-treatment to post-treatment in all variables. Statistical analysis was performed in SPSS version 19.0. The level of significance was set at *p* < 0.05.
